# Altered gut microbiota after traumatic splenectomy is associated with endotoxemia

**DOI:** 10.1038/s41426-018-0202-2

**Published:** 2018-11-30

**Authors:** Hua Zhu, Yang Liu, Shengda Li, Ye Jin, Lei Zhao, Fuya Zhao, Jing Feng, Wei Yan, Yunwei Wei

**Affiliations:** Department of Oncology and Laparoscopy Surgery, the First Affiliated Hospital of Harbin Medical University, 150001 Harbin, China

## Abstract

Splenectomy carries a long-term risk of postoperative infection, and the chronic, low-grade inflammation associated with endotoxemia may be related to the gut microbiota. In this study, to increase our understanding of the potential cause of the high rate of infection in postsplenectomy patients, we evaluated the differences in the gut microbiota and plasma lipopolysaccharide level of patients after splenectomy relative to those of healthy controls. Thirty-two patients having undergone splenectomy and 42 healthy individuals were enrolled into the splenectomy (SP) and healthy control (HC) groups, respectively. The SP group was subdivided into three subgroups according to the length of their postoperative time. Fecal samples were used for gut microbiota analysis via 16s rRNA gene sequencing, blood examinations and plasma lipopolysaccharide measurements were also taken. Significant differences were observed in gut microbiota composition with regard to the relative bacterial abundances of 2 phyla, 7 families, and 15 genera. The lipopolysaccharide level was significantly higher in the SP group than in the HC group and were negatively associated with five bacterial families with low abundance in the SP group. The degree of the microbiota alteration increased with the length of the postoperative time. The PICRUSt analysis showed that the relative abundances of *lipopolysaccharide biosynthesis proteins* and *lipopolysaccharide biosynthesis* pathways were higher in the SP group and were positively associated with the plasma lipopolysaccharide level. Significant alterations were observed in the gut microbiota of the splenectomized patients and were associated with plasma lipopolysaccharide level. Further studies are needed to verify whether such alterations after splenectomy are related to an increased risk of complications.

## Introduction

Splenectomy is an effective therapeutic procedure for spleen rupture subsequent to trauma, but it is not to be undertaken lightly. In each case, the risks are weighed against the potential benefits^1^. However, as many as 30% of patients with spleen injury undergo total splenectomy due to the severity of the injury or technical limitations^2^. An infection rate of 3.2% with a mortality of 1.3% has been reported in splenectomized patients^3^. Furthermore, overwhelming postsplenectomy infections, the most considered complication, have been reported to be a long-term major risk for patients after splenectomy^1^. Overwhelming postsplenectomy infections were first reported by King and Schumacker^4^. These infections include fulminating sepsis, meningitis, or pneumonia in splenectomized and hyposplenic individuals and are caused by *Streptococcus pneumoniae*, *Neisseria meningitidis*, and *Haemophilus influenzae type B*^5^.

Low-grade inflammation is a putative risk factor for infections^6^ and is characteristic of acute and chronic diseases that are associated with elevated circulating lipopolysaccharide (LPS), including sepsis, type II diabetes, and atherosclerosis^7^. LPS is a major component of the cell wall of gram-negative bacteria and has been extensively studied with regard to pathogen-associated molecular patterns in low-grade, chronic inflammation^6^. It has been hypothesized that most plasma LPS is derived from the gut, since the gut microbiota is the biggest source of gram-negative bacteria in the body^8^. In addition, a high-fat western diet and dietary fiber have an effect on intestinal permeability, which can promote and inhibit the translocation of LPS into the blood, respectively^9–11^.

An altered gut microbiota has also been observed in patients with type II diabetes and atherosclerosis, which are accompanied by  low-grade, chronic inflammation and endotoxemia^12^.

The function and composition of the gut microbiota may undergo changes after homeostatic changes occur in the host^13^. In the past decade, the interactions between microbiota and some diseases characterized by low-grade, chronic inflammation have attracted a great deal of attention^14–16^. Gut microbiota may contribute to chronic disease by influencing the immune responses of the host, indicating that it may be implicated in postsplenectomy complications in patients^6^. However, the composition of the gut microbiota in patients after splenectomy has not been investigated.

In the present study, we performed 16s rRNA gene sequencing to characterize the gut microbiota of splenectomized patients and assessed the potential association between the gut microbiota and plasma LPS level. It is our hypothesis that patients who have undergone splenectomy have an altered gut microbiota, which may cause low-grade, chronic inflammation that may contribute to the high risk of postsplenectomy complications.

## Results

### Study population

Thirty-five patients who had undergone splenectomy and met the inclusion criteria were enrolled in this study. Two patients were excluded due to low stool DNA quality while another patient was excluded for chylemia. Finally, 32 patients were enrolled into the splenectomy (SP) group, and 42 volunteers were enrolled into the healthy control (HC) group (Fig. S1). The 1-, 2-, and 3-year subgroups were composed of 12, 9, and 11 patients, respectively. Between the two groups and among the SP subgroups, there were no significant differences in age, gender or body mass index (BMI).

### Gut microbiota alterations after splenectomy

**Fig. 1:**
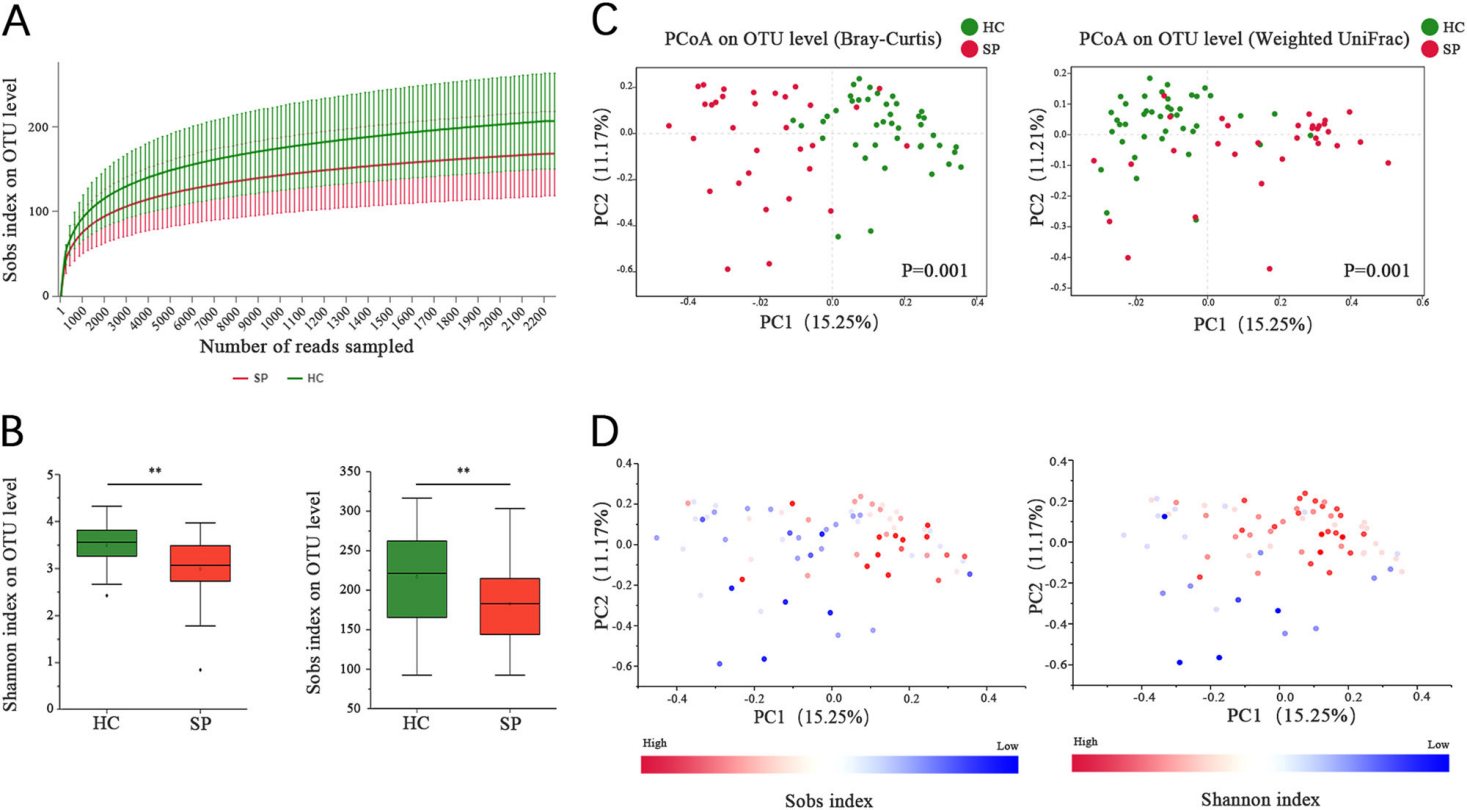
The gut microbiota in splenectomy patients differs from that of healthy controls (**A**) Rarefaction curves were used to compare species richness between the SP and HC groups. (**B**) Wilcoxon’s test shows that the differences in the Sobs and Shannon indices between the SP and HC groups were significant at the OTU level (**P value < 0.01, Wilcoxon rank sum test). (**C**) A principal coordinate analysis (PCoA) for the HC and SP groups, with plots based on both the Bray−Curtis distance (Pr(>F) = 0.001) and Weight-UniFrac distance matrices (Pr(>F) = 0.009, PERMANOVA test). The first two coordinates are plotted with the percentage of variability as indicated on the axis. Each point represents a sample, and the colors represent different groups. (**D**) The Sobs and Shannon indices for each sample are represented by points on a Bray−Curtis distance PCoA, where a blue point represents a low value, a white point represents a mid-value and a red point represent a high value

After filtering, an average of 32,586 reads per sample were obtained (22,085−47,660). The sample size was equalized to 22,085 for each sample using random subtraction. Sequencing depths were examined by plotting the rarefaction curve for richness (Sobs index). All of the samples reached plateaus, suggesting that sequencing depth was adequate (Fig. 1A). The sequences were clustered into 725 operational taxonomic units (OTUs). Within the data, clustering identified the presence of 227 genera within 68 families and 16 phyla. The dominant phyla in the two groups were Firmicutes and Bacteroidetes (Fig. S2).

The alpha diversity of the gut microbiota was assessed using the Shannon and Sobs indices. The Shannon index measures both richness and evenness, while the Sobs index measures only the richness. The Sobs index for the gut microbiota in the HC group (205.71 ± 56.86) was higher than in the SP group (167.28 ± 50.73, *P* = 0.008, Wilcoxon rank sum test). In addition, the Shannon index was higher in the HC group (3.49 ± 0.45) than in the SP group (2.98 ± 0.68, *P* < 0.001, Wilcoxon rank sum test; Fig. 1B).

Beta diversity was assessed using Bray−Curtis and Weighted UniFrac distance metrics and was visualized by principal coordinates analysis (PCoA). The results showed that the overall microbial composition of splenectomized patients deviated from that of the healthy controls using both the Bray−Curtis (PERMANOVA test, Pr(>F) = 0.002) and Weighted UniFrac distance metrics (PERMANOVA test, Pr(>F) = 0.009, Fig. 1C). Notably, the Sobs and Shannon indices can explain the distribution of samples in PCoA based on Bray−Curtis (Fig. 1D). A difference between patients with different postoperative times was also observed with the Bray−Curtis distance-based PCoA (PERMANOVA test, Pr(>F) = 0.029). However, a significant difference was not observed for the Weighted UniFrac distance (PERMANOVA test, Pr(>F) = 0.125, Fig. S3).

### Specific bacteria taxa were associated with splenectomized patients

**Fig. 2:**
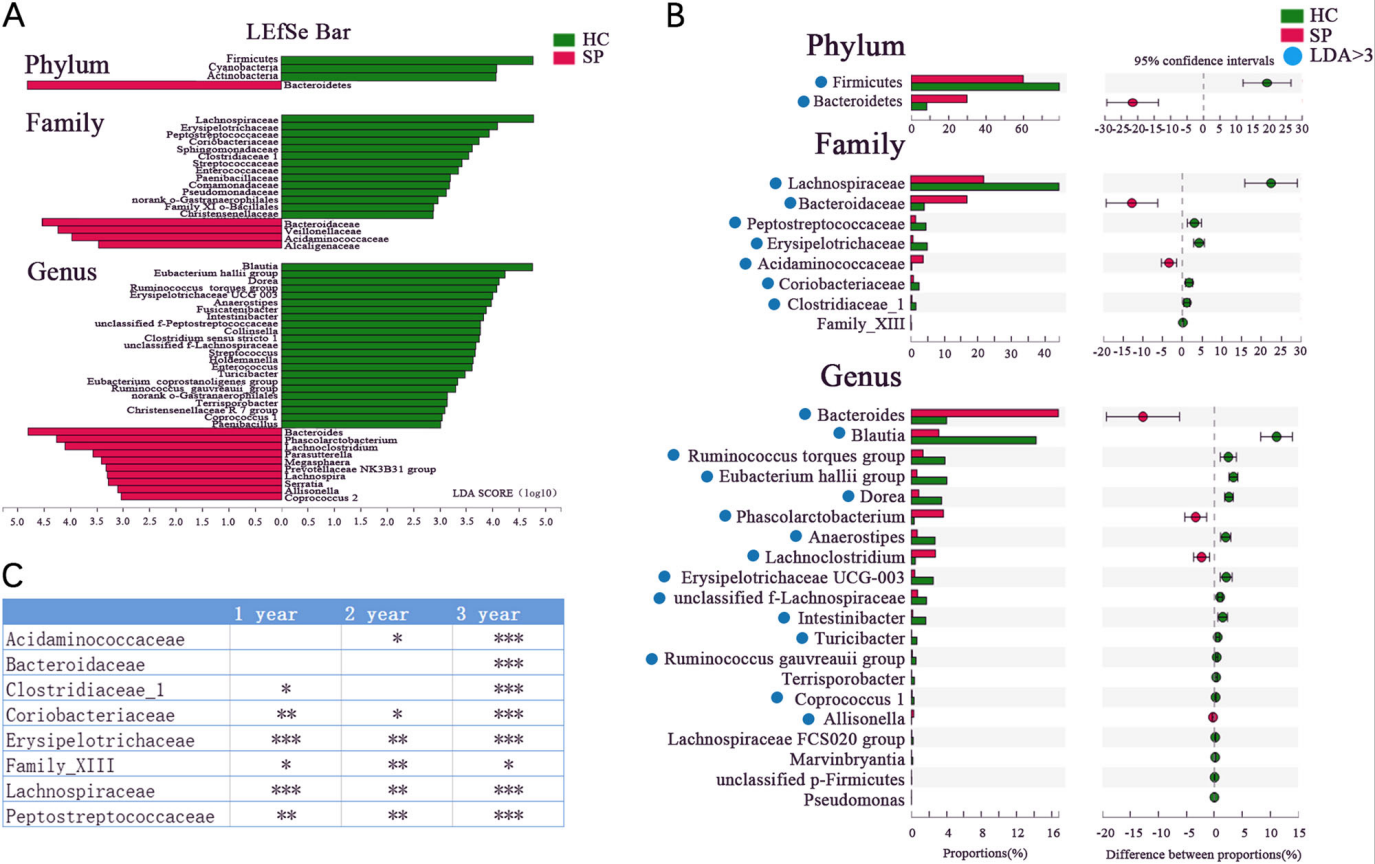
Differences in specific bacterial taxa in splenectomized patients compared to the healthy controls **(A)** Taxonomic cladogram obtained using linear discriminant analysis (LDA) effect size (LEfSe) analysis. LEfSe identified the taxa with the greatest differences in abundance between the SP and HC groups. At the phylum, family, and genus levels, only taxa meeting a significant LDA threshold value of >3 are shown. (**B**) Wilcoxon rank sum test was used for comparisons of the relative abundances at the phylum, family, and genus levels in the SP and HC groups. Only *P*_fdr_ values < 0.05 are shown, and the blue points indicate that the taxa appeared in (**A**). (**C**) Differentially abundant taxa at the family level between 1-, 2-, and 3-year subgroups  and HC group respectively (Only *P*_fdr_ values < 0.05 are shown, *P_fdr_ < 0.05, **P_fdr_ < 0.01, ***P_fdr_ < 0.005)

LDA Effect Size (LEfSe) analysis was performed to detect bacterial taxa with significantly different abundances between the groups (Fig. 2A). The phyla Firmicutes, Cyanobacteria, and Actinobacteria were significantly enriched in the HC group compared to the SP group, while Bacteroidetes was enriched in the SP group. The relative abundances of 18 bacterial families significantly differed between the two groups, with those of 4 and 14 families being enriched in the SP and HC groups, respectively. Furthermore, the relative abundances of 33 genera were significantly different between the two groups, 23 of which were significantly enriched in the HC group compared to the SP group.

The Wilcoxon rank sum test with false discovery rate (FDR) adjustment was used to describe the specific gut microbiota alterations in the SP group. At the phylum level, a lower abundance of the phylum Firmicutes was observed in the SP group compared to the HC group, while that of the phylum Bacteroidetes was higher in the SP group. At the family level, the relative abundances of eight families were observed to be significantly altered, with six families having higher relative abundances in the HC group compared to the SP group (Fig. 2B). Genera with more than 0.1% richness from the splenectomy patients and healthy control were used for the differential analysis. Twenty bacterial genera that displayed different abundances between the SP and HC individuals were observed. Four genera had a higher relative abundance in the SP group, while the 16 other genera had significantly higher relative abundances in the HC group (*P*_fdr_ < 0.05, Wilcoxon rank sum test, Fig. 2B). Of the taxa identified in this analysis, we showed that the 2 phyla, 7 of 8 families and 15 of 20 genera coincided with the LEfSe results (Fig. 2B). In addition, 6, 6, and 8 differently distributed bacterial families were observed between the HC group and the 1, 2, and 3-year subgroups of the SP group, respectively (Wilcoxon rank sum test, *P*_fdr_ < 0.05, Fig. 2C).

### Plasma LPS level were higher in splenectomized patients than the healthy controls and correlated with gut microbiota

**Fig. 3:**
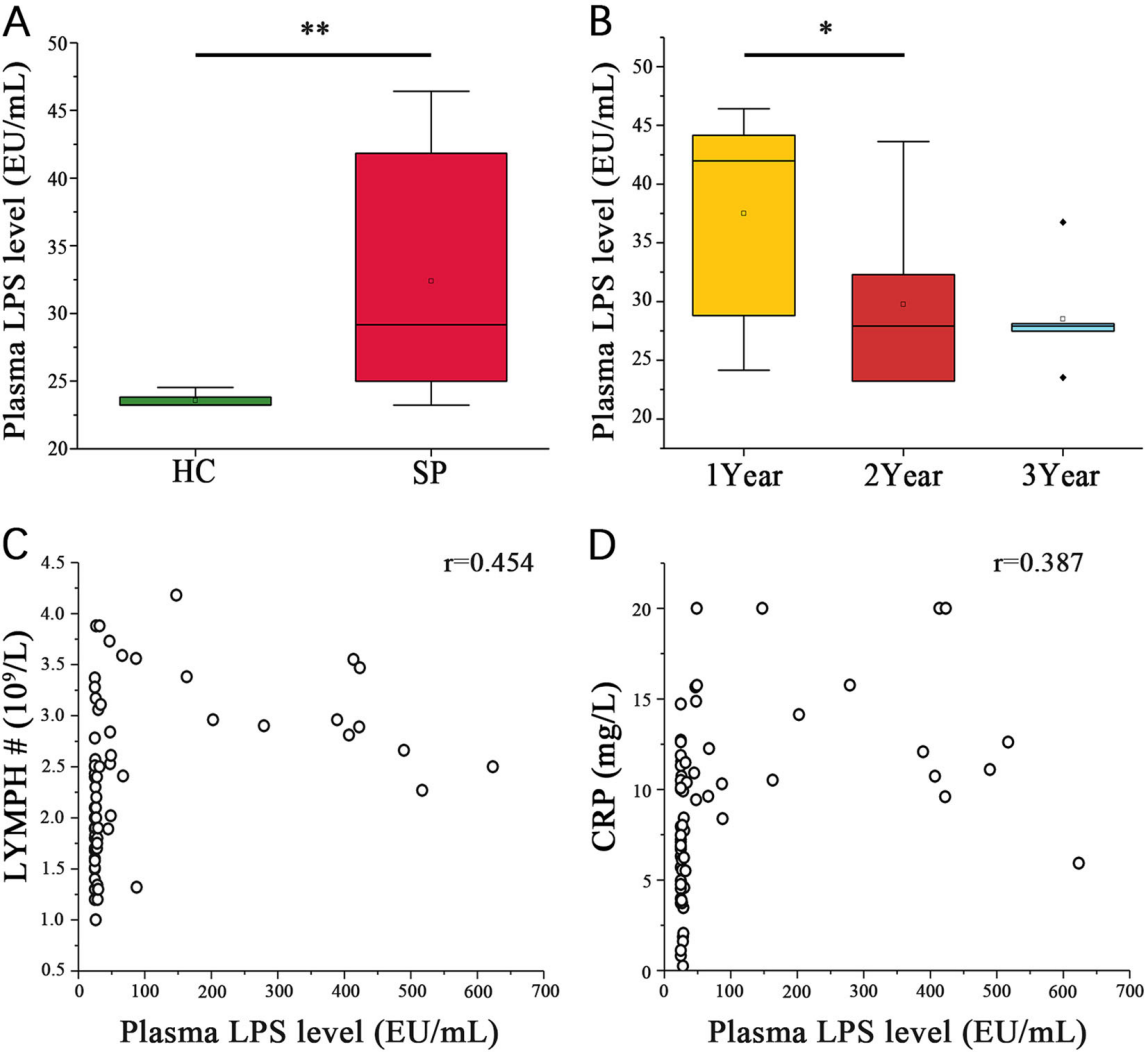
Correlation analysis of blood indicators and plasma LPS level. **(A)** Wilcoxon rank sum test shows that the SP and HC group plasma LPS levels are significantly different. (**B**) Wilcoxon rank sum test shows a significant difference in the plasma LPS levels among the patient subgroups stratified by postsplenectomy time (*P < 0.05, **P < 0.01, Wilcoxon rank sum test). Associations among plasma LPS level and (**C**) LYMPH# or (**D**) plasma CRP level in each sample were estimated using Spearman’s correlation analysis (*r* = 0.454, *P* < 0.05, Spearman’s rank test)

Compared with the HC group, the SP group had significantly higher numbers of white blood cells and platelets and a higher lymphocytes number count (LYMPH #, Table 1). The plasma LPS of the SP group (167.20 ± 184.97 EU/mL) was almost sixfold higher than that of the HC group (26.27 ± 1.72 EU/mL; *P* < 0.001, Wilcoxon rank sum test, Fig. 3A). In the SP subgroups stratified by time after splenectomy, only the plasma LPS level differed significantly, decreasing over time (*P* = 0.036, Kruskal−Wallis test, Fig. 3B). A significant positive correlation was observed between plasma LPS level and LYMPH # (*r* = 0.454, *P* < 0.001, Spearman’s rank test, Fig. 3C). A significantly higher plasma C-reaction protein (CRP) concentration was observed in the SP group (12.2 ± 4.01 mg/L) compared to the HC group (6.49 ± 3.22 mg/L, *P* < 0.001, Wilcoxon rank sum test), but no significant difference was observed for Procalcitonin (PCT, Table 1). In addition, plasma LPS level was positively correlated with the plasma CRP level (*r* = 0.387, *P* < 0.01, Spearman’s rank test, Fig. 3D).

**Table 1:** Basic information and blood examination results

	SP	HC	*P*
Gender, F/M	2/30	5/37	0.245
Age, year	41.22 ± 11.55	45.78 ± 12.02	0.072
BMI, kg/m^2^	23.90 ± 4.49	24.12 ± 4.05	0.461
LPS level, EU	167.20 ± 184.97	26.27 ± 1.72	0.001
CRP level, mg/L	12.2 ± 4.01	6.49 ± 3.22	0.001
PCT level, ng/L	0.032 ± 0.015	0.027 ± 0.015	0.211
WBC, 10^9^/L	7.78 ± 2.23	5.86 ± 1.21	0.001
LYMPH, %	39.59 ± 10.27	32.44 ± 6.25	0.002
LYMPH#, 10^9^/L	2.87 ± 0.64	1.9 ± 0.55	0.000
NEUT#, 10^9^/L	0.46 ± 0.21	0.42 ± 0.14	0.167
RBC, 10^12^/L	4.82 ± 0.53	4.99 ± 0.42	0.236
PLT, 10^9^/L	404.02 ± 95.37	240.04 ± 48.17	0.001

*PCT* procalcitonin, *LYMPH* lymphocyte, *NEUT* neutrophils, *PLT* platelet count, *RBC* red blood cell, *WBC* white blood cell

**Fig. 4:**
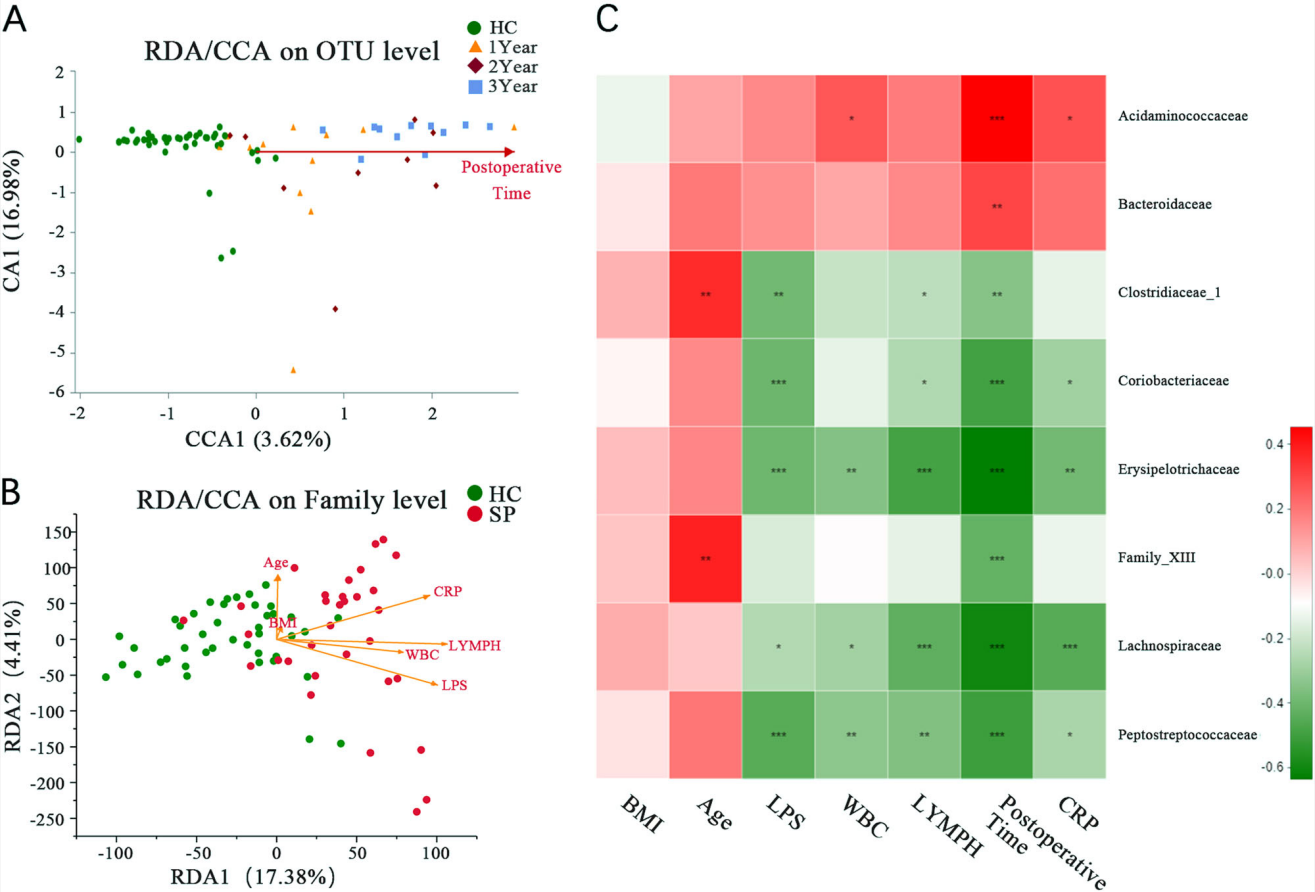
Correlation analysis of clinical parameters and the gut microbiota. (**A**) CCA shows the composition of the gut microbiota is altered as the increasing postoperative time after splenectomy at the OTU level. (**B**) CCA shows the clinical parameters (including plasma LPS level, WBC, LYMPH#, BMI, CRP, and age) associated with the gut microbiota composition at the OTU level and associated with each other. (**C**) Association among seven clinical parameters and the relative abundances of eight families with altered abundances (see Fig. 2B) in the SP and HC groups were estimated using Spearman’s correlation analysis using Heatmap. Color intensity represents the magnitude of correlation. Red, positive correlations; green, negative correlations. *P value < 0.05; **P value < 0.01; ***P value < 0.001

Canonical correspondence analysis (CCA) and redundancy analysis (RDA) were similar to the PCoA result but were constrained to ordination by environmental factors to determine how the microbiota data were distributed along environmental gradients. We performed CCA to assess whether the distribution of samples was related to factors other than splenectomy. The results showed that the degree of microbiota alteration became notable as the postsplenectomy postoperative time increased (Fig. 4A). RDA showed that the plasma LPS level, CRP concentration, white blood count (WBC), and LYMPH# were positively correlated with the gut microbiota, with LPS being the most correlated. BMI and age were not significantly related to gut microbiota (Fig. 4B).

To assess the association between the clinical parameters and bacterial taxa, a correlation analysis was conducted among seven clinical parameters (BMI, Age, LPS, CRP, WBC, LYMPH# and Postoperative time), first with eight bacterial families and then with 20 genera, which were differentially distributed between the HC and SP groups. The results were visualized by Heatmap. Plasma LPS was observed to be negatively associated with five of the eight bacterial families (*P* < 0.05, Spearman’s rank test, Fig. 4C). The abundances of all five families were lower in the SP group than in the HC group (Table S2). Ten of the 20 genera were significantly negatively associated with the plasma LPS level (*P* < 0.05, Spearman’s rank test). The relative abundance of *Lachnoclostridium* was positively correlated with the plasma LPS level (*r* = 0.276, *P* = 0.023, Spearman’s rank test, Fig. S4), and its relative abundance was significantly higher in the SP group (2.67 ± 3.7) than in the HC group (0.44 ± 0.55, *P* < 0.001, Wilcoxon rank sum test). In contrast, the relative abundance of *Intestinibacter*, for which the community abundance was significant lower in the SP (0.17 ± 0.29) group than in the HC group (1.67 ± 2.81, *P* < 0.001, Wilcoxon rank sum test), showed the strongest negative correlation with the plasma LPS level (*r* = –0.455, *P* < 0.001, Spearman’s rank test, Fig. S4).

Because most bacterial families exhibit a specific gram staining reaction, we compared the gram staining reactions for all 68 bacterial taxa at the family level. Twenty-nine gram-negative families were identified, and the percentage of the community abundance of gram-negative bacteria in the SP group (39.77 ± 19.37%) was almost 2.5-fold higher than that of the HC group (15.40 ± 12.04%, *P* < 0.001, Wilcoxon rank sum test, Fig. S5). In addition, plasma LPS also positively and significantly correlated with the relative community abundance of gram-negative bacteria (*r* = 0.314, *P* < 0.001, Spearman’s rank test, Fig. S6), indicating that high proportion of gram-negative bacteria may be associated with high plasma LPS level in splenectomized patients.

**Fig. 5:**
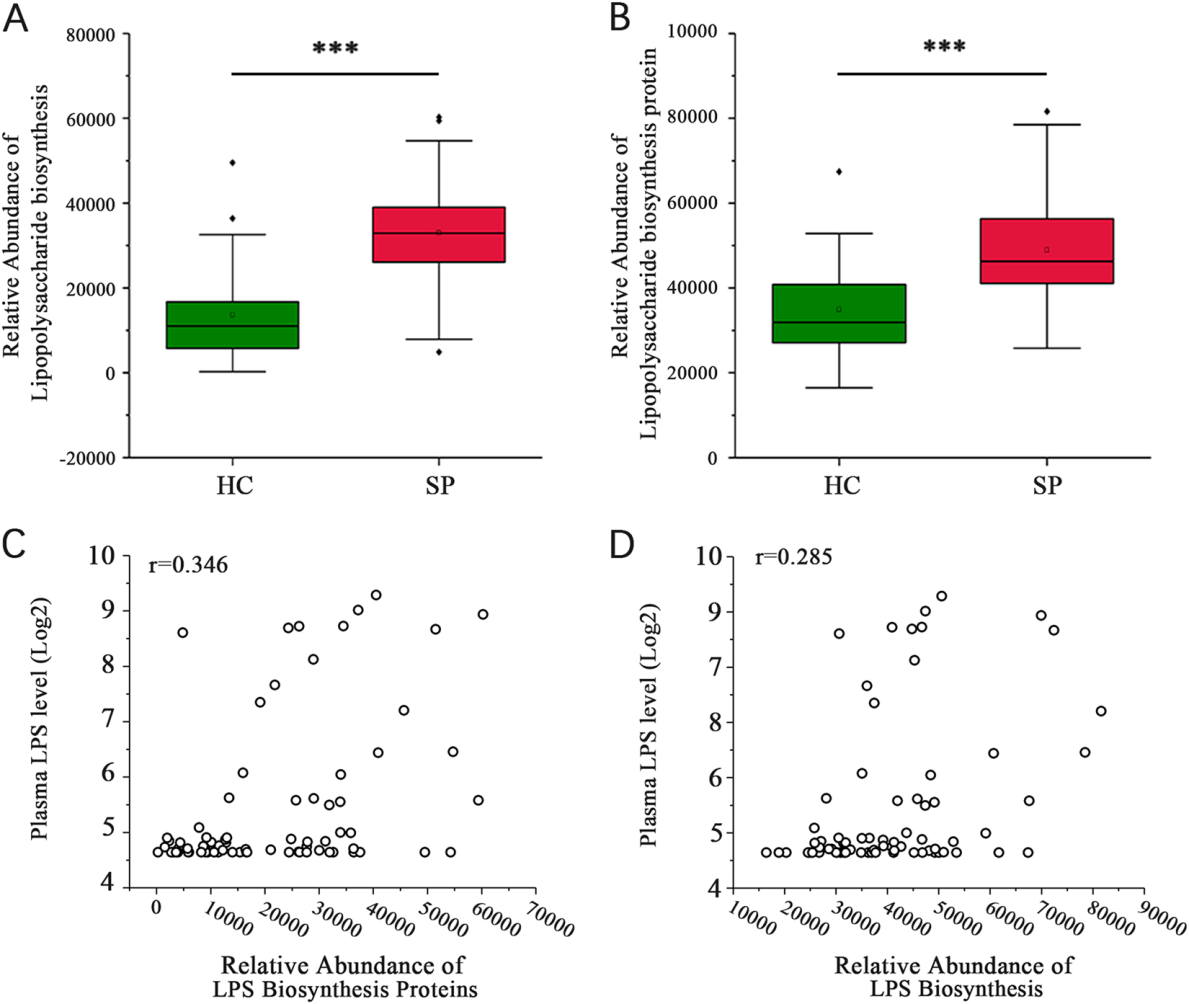
Expression of LPS-associated pathways. (**A**, **B**) Significant differences in the relative abundances of the *lipopolysaccharide biosynthesis* and *lipopolysaccharide biosynthesis protein* pathways between the SP and HC groups (***P < 0.001, Wilcoxon rank sum test). (**C**) The *lipopolysaccharide biosynthesis proteins* pathway is associated with the plasma LPS level (*r* = 0.346, *P* = 0.003, Spearman’s rank test). (**D**) The *lipopolysaccharide biosynthesis* pathway is associated with the plasma LPS level (*r* = 0.285, *P* = 0.014, Spearman’s rank test)

Biofunctions of the gut microbiota were predicted from 16s rRNA gene sequencing data using PICRUSt analysis. An analysis of KEGG pathway categories (level III) showed that the number of reads in two LPS-related pathways, *lipopolysaccharide biosynthesis* and *lipopolysaccharide biosynthesis proteins*, were higher in the SP group than in the HC group (32,929.87 ± 13,640.79 vs. 13,661.98 ± 11,046.56; 48,910.62 ± 13,852.9 vs. 34,897.19 ± 10,359.79, *P* < 0.001, respectively, Wilcoxon rank sum test, Fig. 5A, B). The plasma LPS level positively correlated with both *lipopolysaccharide biosynthesis proteins* (*r* = 0.346, *P* = 0.003 Spearman’s rank test, Fig. 5C) and *lipopolysaccharide biosynthesis* (*r* = 0.285, *P* = 0.014, Spearman’s rank test, Fig. 5D).

## Discussion

We performed a cross-sectional study to examine the differences in the gut microbiota and plasma LPS level of patients after splenectomy relative to healthy controls. The splenectomized patients exhibited an altered gut microbiota composition and function and significantly higher level of plasma LPS relative to the healthy control group. A higher abundance of gram-negative bacteria was observed in the gut microbiota of splenectomized patients compared with the healthy controls, indicating that the altered gut microbiota composition was a major cause of the elevated plasma LPS in the splenectomized patients. CCA showed the degree of microbiota alteration increased with postoperative time.

Few studies have investigated alterations in the gut microbiota of patients after traumatic splenectomy. In the present study, splenectomized patients’ gut microbiota exhibited lower alpha diversity, which has also been described for some other diseases and indicates a poor gut microbiota status. Ott et al. reported a reduction in the colonic microflora diversity of Crohn’s disease patients^17^, and de la Cuesta-Zuluaga et al. reported a reduction in alpha diversity in obese individuals^18^. A lower alpha diversity has also been reported to be associated with some other diseases, such as graft-versus-host disease^19^ and liver cirrhosis^20^, with significant alterations were observed for different bacterial taxa. Similar alterations have been reported to be associated with other diseases. For example, the abundances of the families *Lachnospiraceae* and *Coriobacteriaceae*, which were shown to be significantly lower in the SP group than in the HC group in the present study, were also reported to be lower in chronic hepatitis B patients^21^. Luedde et al^22^. observed a lower abundance of *Erysipelotrichaceae* in patients with chronic heart failure, and another study observed that a lower abundance of *Bacteroidaceae* was correlated with obesity^23^. The family *Bifidobacteriaceae*, which was significantly reduced in the 2-year subgroup (Data did not show), is a well-known probiotic that has been reported to improve metabolic endotoxemia and impaired glucose tolerance^24^.

Identifying the mechanism for gut microbiota variation postsplenectomy was not the primary purpose of this study, and we speculated that immunodeficiency in these patients could lead to this outcome. The spleen is a secondary lymphoid organ that can supply the intestine with immune cells, promote the immune response against invading pathogens and maintain gut microbiota homeostasis. Several studies have shown that the spleen contributes to the mucosal immune response in the gut^25,26^, as there is a “highway” between the human splenic and gut mucosal marginal zones^27^. The sIgA film that lines the intestinal epithelium, which plays a role in regulating the commensal microflora and in preventing pathogen invasion, is derived from B-1a cells that differentiate from lineage-negative precursor cells that are present exclusively in the spleen^28,29^. Thus, there is reason to believe that the abnormal gut mucosal immune microenvironment of patients without a spleen cannot maintain the normal gut microbiota.

In the SP group, 26 of the 32 patients reported feeling a lower activity endurance; 13 patients reported being susceptible to catching colds; and 17 patients had been administered antibiotics more than 3 months prior to sample collection after splenectomy. Additional PCoA analysis showed patients’ gut microbiota were not significantly different on account of the condition of susceptible to catch cold or not, and antibiotics application history or not (data did not show). These results indicate that infectious and antibiotic application history of patients were not the factor associated with microbiota composition in this study. A study observed that the degree of microbial dysbiosis increases with age and fuels inflammation^30^. However, in the present study, the CCA showed that age was not associated with microbiota composition. The small age range of participants in this study may have given rise to this inconsistent result. The plasma LPS and CRP levels and the WBC count and LYMPH# showed notable correlations with the microbiota composition, indicating a relationship between the gut microbiota and low-grade, chronic inflammation.

Because release of LPS is a consequence of the lysis of gram-negative bacteria^31^, another hypothesis is that the gut is the source of most plasma LPS, since the gut microbiota is the largest reservoir of gram-negative bacteria in the body^6^. The relative abundance of gram-negative bacteria accounted for 15.40% of the bacterial abundance in the HC group, but up to 39.77% in the SP group. Furthermore, the predicted microbiota functions determined by the KEGG pathway analysis indicated a positive correlation between the two enriched pathways (*lipopolysaccharide biosynthesis* and *lipopolysaccharide biosynthesis protein*) and plasma LPS in the patients. This result supports the hypothesis that the altered gut microbiota in the splenectomized patients may have led to active LPS metabolism in the gut, resulting in the subsequent translocation of the LPS across the intestinal barrier and its mildly increased concentration in blood. Taken together, these results suggest that the gut microbiota may contribute to high plasma LPS in patients after splenectomy.

Low-grade or metabolic endotoxemia refers to subclinically elevated level of plasma LPS^32^. Endotoxemia has been shown to be a pathogen-associated molecular pattern for low-grade, chronic inflammation. Furthermore, endotoxemia has been observed to lead to low-grade, chronic inflammation due to some chronic infections, adverse lifestyles and advanced age^6^. A mild increase in plasma LPS level and low-grade, chronic inflammation was also observed in noncommunicable diseases, such as type II diabetes^33^, atherosclerosis^34^, chronic fatigue syndrome^35^ and depression^36^. The elevated plasma LPS level observed in splenectomized patients persists in the long term, which can lead to a state of low-grade, chronic inflammation that alters the innate immune environment, which may lead to a high risk of infection and chronic diseases. A study of an Italian cohort observed that compared with subjects with a plasma LPS level of <250 EU/mL, those with higher level had a threefold increased risk of atherosclerosis, especially smokers^37^. In the present study, nine patients reached a plasma LPS level of 50 pg/mL. Whether these patients are at high risk of cardiovascular disease or other chronic diseases requires long-term observation.

The gut microbiota may be a potential target to prevent endotoxemia. Recent studies have shown the effects of antibiotics and prebiotics on the gut microbiota for various diseases. Antibiotics are the traditional choice for patients with endotoxemia, although the positive effects of this treatment are not definitive. Antibiotic treatment of fructose-induced fatty liver mice markedly ameliorated endotoxemia^38^. In a study by Kalambokis and Tsiano^39^, the antibiotic rifaximin reduced endotoxemia and improved liver function. However, the results of recent studies have shown that dietary fiber and prebiotics/probiotics can be used to improve intestinal integrity and reduce signs of endotoxemia in mice^10,11,40,41^. Thus, administration of antibiotics and probiotics targeting the gut microbiota may be a promising method of treating diseases caused by endotoxemia.

Previous studies have focused on the complications of splenectomy, although little attention has been given to changes in the gut microbiota of patients after splenectomy. In the present study, we explored the low-grade, chronic inflammation status of splenectomized patients associated with changes in the gut microbiota. This study had a few limitations. First, because an investigation of the gut barrier function was not included, it is unknown whether the altered gut microbiota leads to an injury of the gut barrier that ultimately causes the observed endotoxemia. Second, the results of a cross-sectional study can be affected by individual differences in gut microbiota, which could be addressed by performing additional studies with more participants. Lastly, the follow-up time used in this study was not long enough to reveal if the altered microbiota and high plasma LPS level are associated with the high infection rate and chronic disease occurrence in splenectomized patients.

In summary, a high percentage of the patients after splenectomy were observed to exhibit endotoxemia that was accompanied by a gradual alteration of gut microbiota over time. Endotoxemia related to gut alterations after splenectomy may lead to a low-grade, chronic inflammation and the onset of complications and disease progression. Therefore, altered gut microbiota should be considered to be a risk factor for splenectomy-related complications. Further study will determine if the altered microbiota composition that occurs after splenectomy has any effect on the health of patients.

## Methods

The Research Ethics Committee of the First Affiliated Hospital of Harbin Medical University approved this clinical trial. The study was performed strictly in accordance with international guidelines regarding the conduct of clinical trials, and each patient provided written informed consent. This study was registered at ClinicalTrials.gov and began on December 20, 2016 (NCT03420599).

### Study population and samples collection

This was a cross-sectional study consisting of two groups, splenectomy patients (SP) and healthy controls (HC). Three subgroups of patients were set according to the postoperative time after the splenectomy, where patients with 1, 2, and 3 years after splenectomy were named the 1-, 2-, and 3-year subgroups. The enrollment of patients who had undergone splenectomies was conducted between January 2017 and January 2018. Enrolled individuals had undergone a total splenectomy for closed abdominal trauma at the First Affiliated Hospital of Harbin Medical University between January 2015 and December 2017. Individuals were excluded from the study for serious abdominal injury of the liver, kidney, or other organs or intestinal rupture.

The healthy control group consisted of individuals who visited the First Affiliated Hospital of Harbin Medical University for routine check-ups. The healthy control subjects and splenectomy patients were well matched with respect to age, gender, and BMI. Individuals were excluded who had a history of surgery, past or present digestive disease (e.g., tumor, irritable bowel, intra-abdominal tuberculosis, or celiac disease), or other systemic disease (hypertension, diabetes, or heart disease). None of the subjects had used any antibiotics or probiotics within the preceding 3 months before fecal sample collection. Each participant was asked to provide a fresh stool sample that was collected in the hospital for gut microbiota analysis. Blood samples were collected for complete blood count and plasma LPS measurements.

### Plasma endotoxin and cytokine examination

Blood samples were drawn into tubes containing Ethylenediaminetetraacetic acid and centrifuged instantly thereafter. Plasma was stored at −80 ℃ until analyzed. The plasma concentrations of IL-1, IL-6, IL-10, TNF alpha, IFN-γ, and CRP were measured using an ELISA Kit (BOSTER, Wuhan, China) according to the manufacturer’s instructions. The plasma PCT level was measured using an Elecsys BRAHMS PCT kit (Roche Diagnostics, Indianapolis, USA). The plasma LPS concentrations were quantified using a chromogenic assay (Gold Mountain River EKT-1M kit, Beijing, China). The samples were analyzed in duplicate against standards of a known analyte dilution, and the mean concentrations were calculated from these measurements for each sample.

### 16s rRNA gene sequencing and bioinformatic analysis

The human fecal samples were collected into 1.5-mL sterile tubes and microbial DNA was extracted using an E.Z.N.A.® soil DNA kit (Omega Bio-Tek, Norcross, GA, USA). The V3−V4 hypervariable regions of the bacterial 16s rRNA gene were amplified. Purified amplicons were pooled in equimolar concentrations and paired-end sequenced (2 × 300) on an Illumina MiSeq platform (Illumina, San Diego, USA) in accordance with standard protocols recommended by Majorbio Bio-Pharm Technology (Shanghai, China).

After filtering the raw reads and conducting quality control, OTUs were clustered with a 97% similarity cut-off using UPARSE (version 7.1, http://drive5.com/uparse/). Chimeric sequences were then identified and removed using UCHIME. Taxonomic assignments for the 16s rRNA gene sequences were made using the RDP Classifier algorithm (http://rdp.cme.msu.edu/) with the Silva (SSU123) 16s rRNA gene database at a confidence threshold of 70%.

Alpha diversity (based on OTU number and Shannon index) and beta diversity (Bray−Curtis and weighted UniFrac distance) were calculated using QIIME (Version 1.7.0) and presented using the ggplot2 package in R. Cluster analysis was preceded by PCoA, which was performed to reduce the dimension of the original variables, using the FactoMineR and ggplot2 packages in R. PERMANOVA of the distance matrices, as implemented in the vegan package in R, was used to identify whether the case/control status explained the significant variation in microbial community composition. The microbiota features differentiating the fecal microbiota were characterized using the linear discriminant analysis LEfSe method for biomarker discovery, which emphasizes both significance and biological relevance. Based on a normalized relative abundance matrix, LEfSe uses the Kruskal−Wallis rank sum test to detect features with significantly different abundance levels between assigned taxa and performs an LDA to estimate the size of the effect for each feature^42^. CCA and RDA were performed using the Multivariate Statistical Package (Kovach, Wales, UK) using normalized environmental variables were normalized. Spearman’s rank test was used for the environmental parameter correlation analysis of bacterial taxa. A heatmap was generated to present the results using the pheatmap package in R. Functional composition profiles of the gut metagenomes were predicted from 16s rRNA gene sequences using PICRUSt with level III KEGG database pathways^43^.

### Statistical analysis

Statistical analysis of the data was performed using SPSS (SPSS version 19, La Jolla, CA, USA). Statistical analysis of continuous data was performed using the Wilcoxon rank sum test. Multiple hypothesis tests were adjusted using the Benjamini and Hochberg FDR, significant differences were considered when the results were below an FDR threshold of 0.05. The Kruskal−Wallis test was used to compare differences between subgroups. Spearman’s rank test was used for correlation analysis. A *P* value of less than 0.05 was considered significant.

## Electronic supplementary material

Supplement material
