# Supplementary material for: Altered gut microbiota after traumatic splenectomy is associated with endotoxemia
Source: Emerg Microbes Infect. 2018 Nov 30;7:197. doi: 10.1038/s41426-018-0202-2 (PMC6265257; doi:10.1038/s41426-018-0202-2)
Supplement: Supplementary file 1 — Supplement material [file 41426_2018_202_MOESM1_ESM.doc]

**Supplemental Information Inventory**

**Figure S1 - Flow chart of participants**

**Figure S2 - Associated with Figure 1**

**Figure S3 - Associated with Figure 2**

**Figure S4 - Associated with Figure 4**

**Figure S5 - The relative abundance of Gram-negative bacteria in study subjects**

**Figure S6 - Associated with Figure 5**

**Table S1 - Clinical data of study subjects**

**Table S2 - bacteria taxa on phylum, family, and genus level Associated with**

**
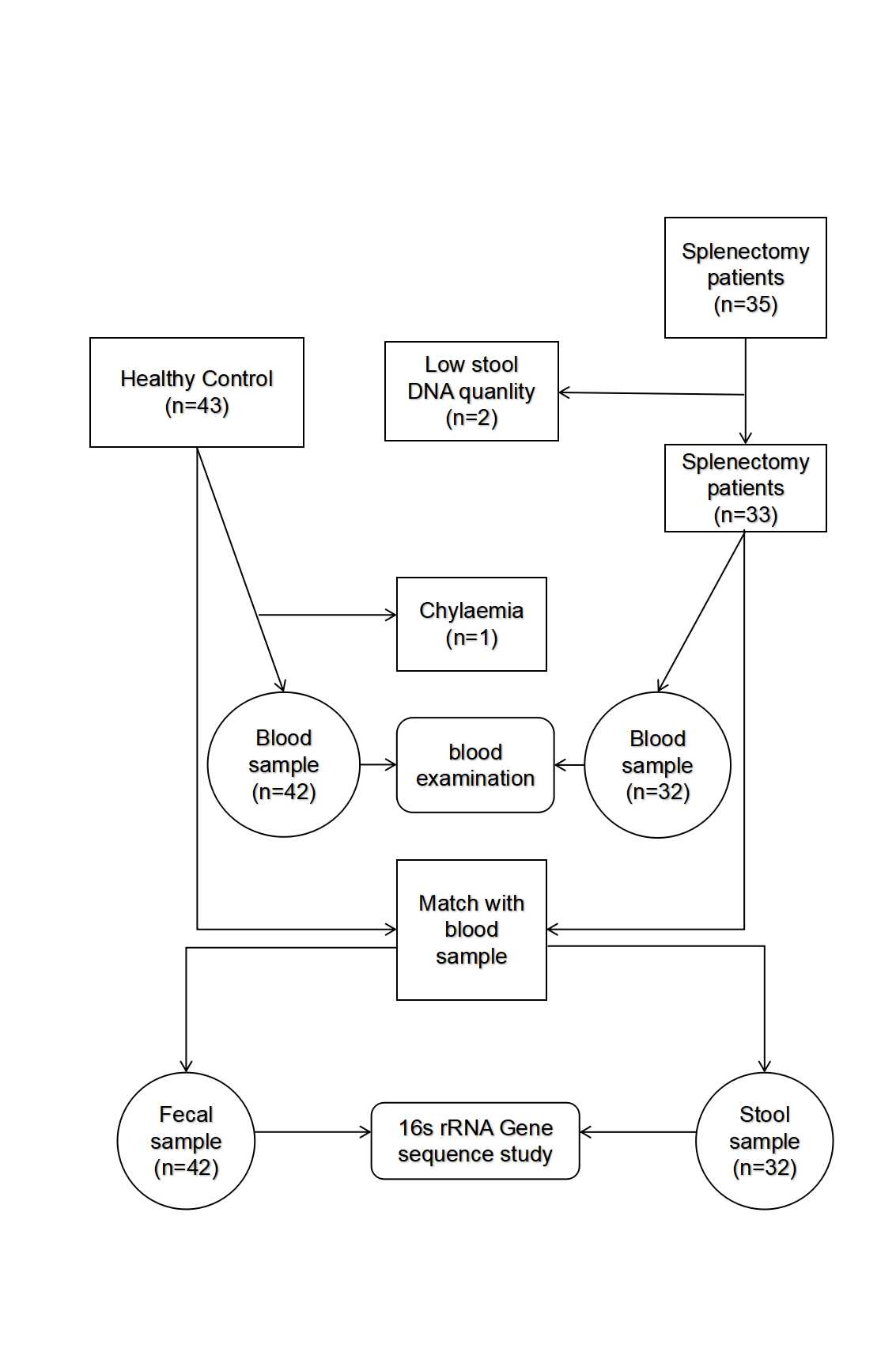
**

**Figure S1.** Flow chart of participants and sample collecting.


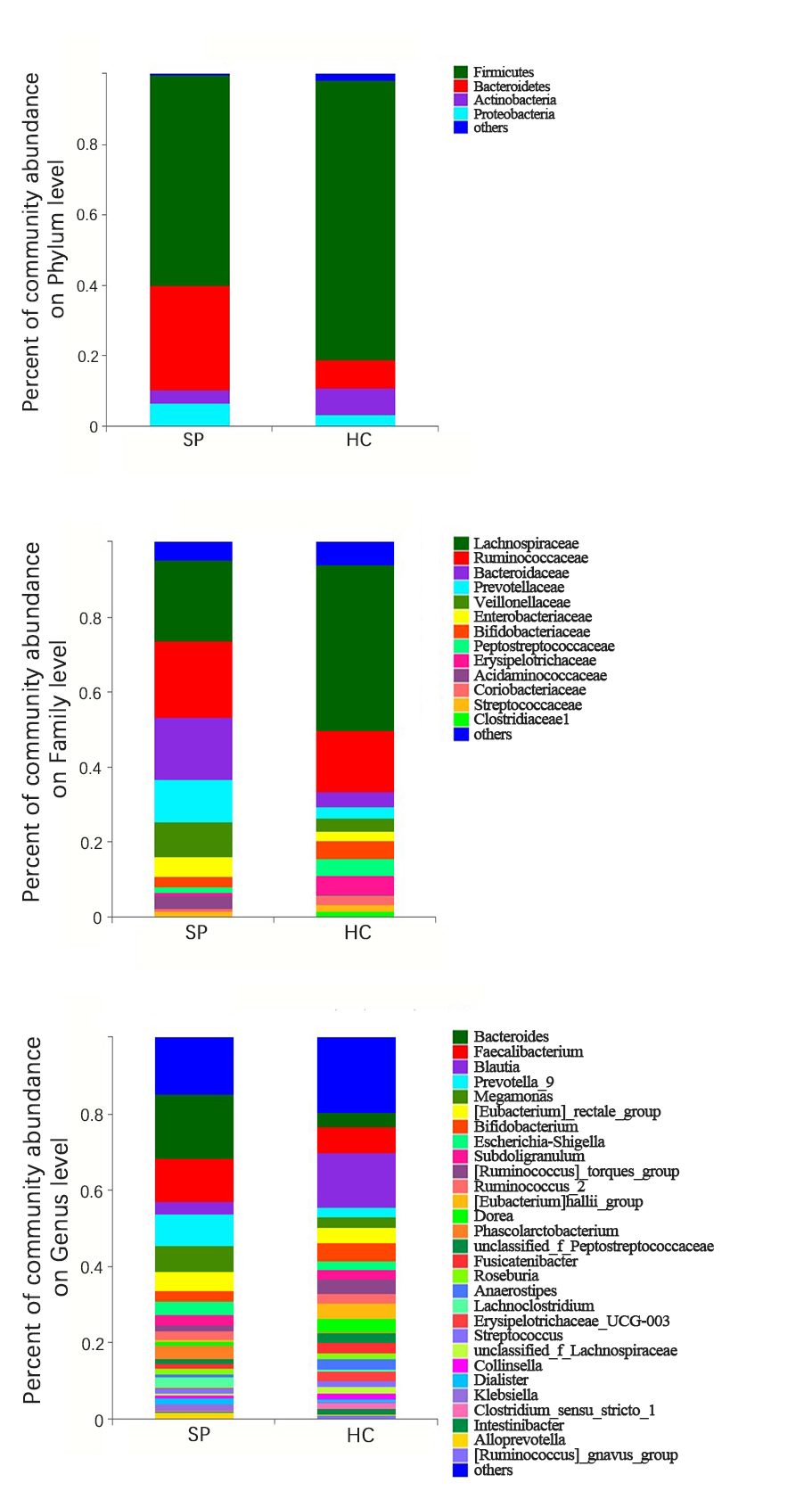


**Figure S2.** Relative abundance of fecal bacteria in the SP and HC groups, on level of phylum, family, and genera. Only taxa with relative abundances of >0.1% were included. All OTUs of lower abundance are grouped as “others”.


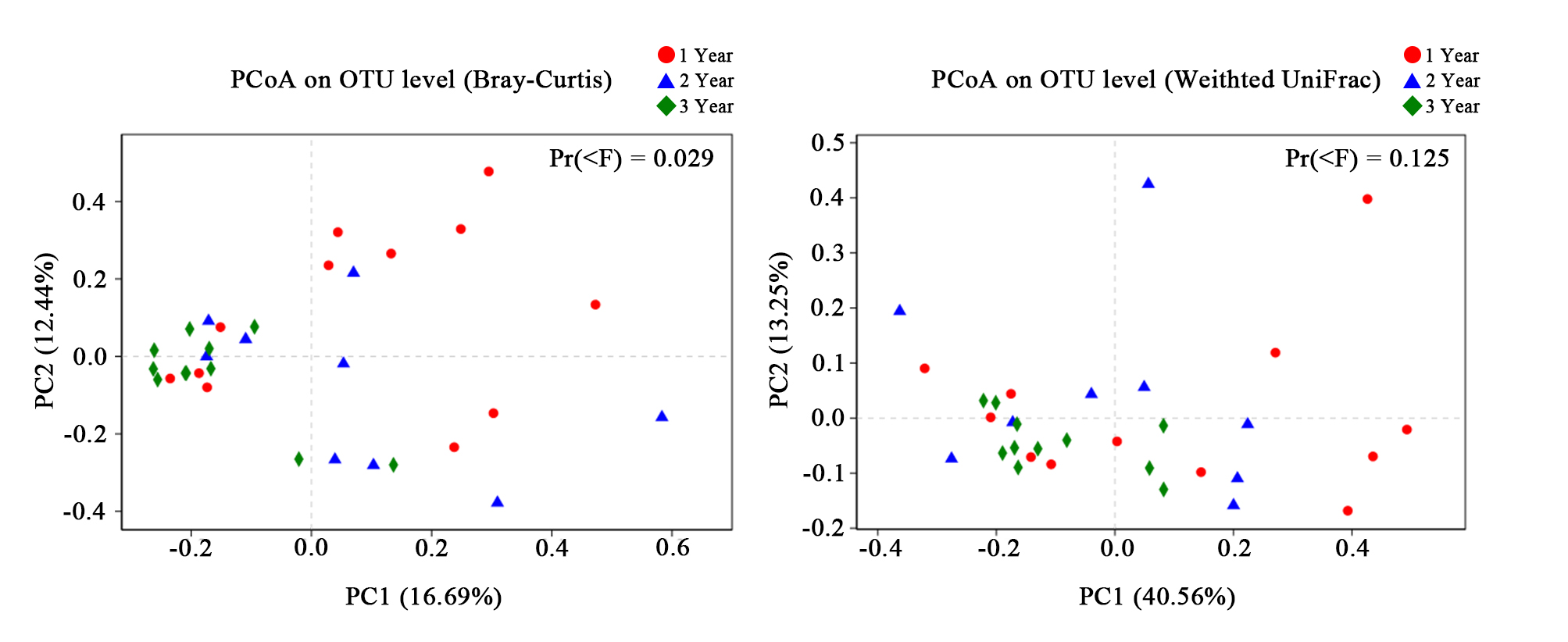


**Figure S3.** PCoA for SP patients with different postoperative time (1,2 and 3 years), plot based on both Bray-Curtis distance and Weighted-UniFrac distance matrices.


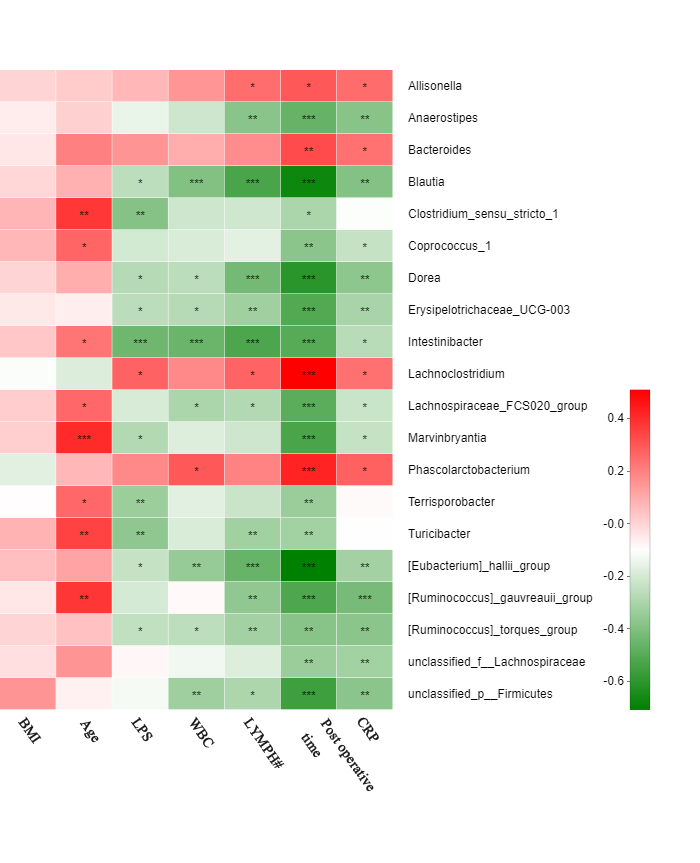


**Figure S4.** Associations among 7 clinical factors and the relative abundance of the 20 significant altered genera in SP and HC groups. Estimations are the results of Spearman’s correlation analysis. Color intensity represents magnitude of correlation. Red, positive correlation; blue, negative correlation. * P-value < 0.05; ** P-value < 0.01; *** P-value < 0.001.


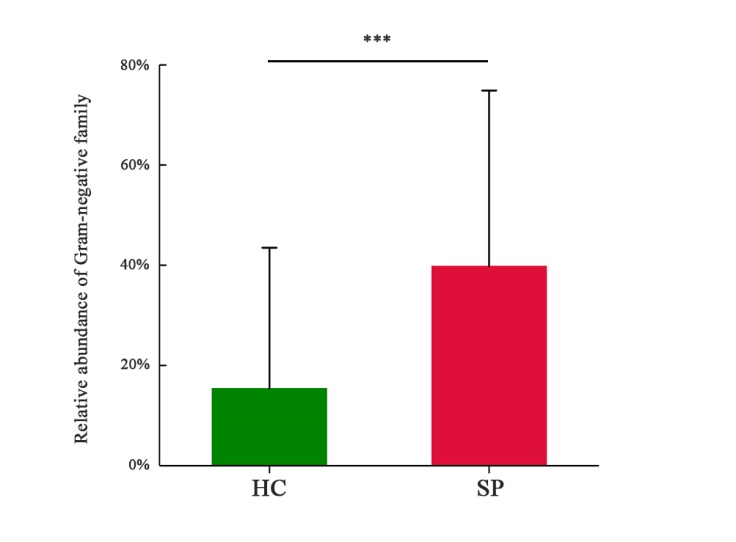


**Figure S5.** The relative abundance of Gram-negative bacteria in the SP group and HC groups.


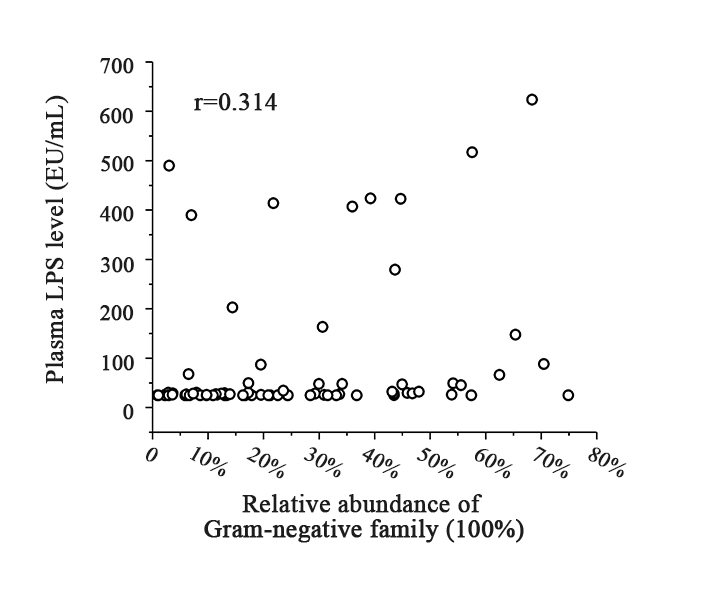


**Figure S6.** Associations among plasma LPS and the relative community of Gram-negative bacteria in each sample (r = 0.314, P < 0.001, spearman’s rank test).

| **Table S1.** Clinical data of study subjects. | | | | | | | | | | | | |  |  | |  |
| --- | --- | --- | --- | --- | --- | --- | --- | --- | --- | --- | --- | --- | --- | --- | --- | --- |
| Patient ID | Group | Agey | BMI  kg/m^2^ | Gender | LPS EU/mL | CRP  mg/L | PCT  ng/mL | WBC  10^9^/L | LYMPH% | NEUT#  10^9^/L | PLT 10^9^/L | Postoperative time y | Activity  endurance | | Antibodies  application history | Infection  frequency |
| Z1 | HC | 40 | 16.98 | Female | 25 | 12.72 | 0.049 | 5.7 | 27.6 | 3.7 | 192 | NA | NA | NA | | NA |
| Z2 | HC | 47 | 23.39 | Female | 25 | 7.47 | 0.018 | 4.7 | 39.8 | 2.2 | 207 | NA | NA | NA | | NA |
| Z3 | HC | 46 | 19.1 | Female | 25 | 6.91 | 0.052 | 6.5 | 29.1 | 3.6 | 261 | NA | NA | NA | | NA |
| Z4 | HC | 39 | 25.98 | Female | 25.65 | 10.27 | 0.027 | 5.5 | 34.8 | 3 | 292 | NA | NA | NA | | NA |
| Z5 | HC | 45 | 38.86 | Female | 26.15 | 10.18 | 0.022 | 4.7 | 36.4 | 2.5 | 178 | NA | NA | NA | | NA |
| H0F01 | HC | 63 | 19.53 | Male | 25 | 6.72 | 0.008 | 5.93 | 25.5 | 3.76 | 188 | NA | NA | NA | | NA |
| H0F04 | HC | 64 | 23.34 | Male | 25 | 6.83 | 0.033 | 5.9 | 22.8 | 4.1 | 219 | NA | NA | NA | | NA |
| H0F10 | HC | 49 | 22.32 | Male | 25 | 3.72 | 0.03 | 5.4 | 43.9 | 2.4 | 273 | NA | NA | NA | | NA |
| H0F17 | HC | 59 | 21.72 | Male | 29.45 | 8.42 | 0.051 | 5.9 | 22.8 | 4.1 | 219 | NA | NA | NA | | NA |
| H0M05 | HC | 30 | 24.8 | Male | 28.15 | 1.86 | 0.019 | 6.7 | 36.6 | 3.4 | 223 | NA | NA | NA | | NA |
| H0M06 | HC | 61 | 26.23 | Male | 25 | 6.81 | 0.032 | 4.67 | 36.04 | 2.42 | 253.4 | NA | NA | NA | | NA |
| H0M11 | HC | 64 | 28.41 | Male | 25.55 | 11.54 | 0.041 | 4.36 | 42.04 | 2.3 | 237 | NA | NA | NA | | NA |
| H0M12 | HC | 45 | 25.71 | Male | 25 | 6.31 | 0.034 | 7.19 | 26.34 | 4.69 | 225.4 | NA | NA | NA | | NA |
| H0M13 | HC | 53 | 22.98 | Male | 25 | 12.63 | 0.04 | 7.95 | 30.6 | 5.2 | 213 | NA | NA | NA | | NA |
| H0M14 | HC | 46 | 25.01 | Male | 28.65 | 2.06 | 0.02 | 4.53 | 29.6 | 2.97 | 249 | NA | NA | NA | | NA |
| H0M15 | HC | 39 | 22.28 | Male | 25.75 | 4.85 | 0.034 | 6.37 | 40.34 | 3.35 | 249.4 | NA | NA | NA | | NA |
| H0M16 | HC | 46 | 21.77 | Male | 25 | 10.02 | 0.046 | 6.22 | 25.4 | 4.35 | 155 | NA | NA | NA | | NA |
| N100 | HC | 43 | 23.21 | Male | 29.25 | 11.57 | 0.03 | 4.1 | 30 | 2.4 | 282 | NA | NA | NA | | NA |
| N102 | HC | 50 | 30.08 | Male | 26.15 | 7.79 | 0.01 | 4.1 | 23.5 | 2.7 | 216 | NA | NA | NA | | NA |
| N103 | HC | 36 | 17.27 | Male | 25 | 5.7 | 0.004 | 5.3 | 28.3 | 3.2 | 344 | NA | NA | NA | | NA |
| N104 | HC | 61 | 26.61 | Male | 25 | 6.7 | 0.029 | 7 | 30.3 | 4.4 | 247 | NA | NA | NA | | NA |
| N105 | HC | 61 | 31.04 | Male | 25 | 0.82 | 0.056 | 6.1 | 27.2 | 3.8 | 179 | NA | NA | NA | | NA |
| N107 | HC | 59 | 28.73 | Male | 29.95 | 4.58 | 0.005 | 4.6 | 28.7 | 2.8 | 155 | NA | NA | NA | | NA |
| N108 | HC | 24 | 20.94 | Male | 28.8 | 3.47 | 0.024 | 6.1 | 29.4 | 3.7 | 195 | NA | NA | NA | | NA |
| N109 | HC | 59 | 23.03 | Male | 25 | 4.52 | 0.004 | 6 | 41.7 | 2.7 | 256 | NA | NA | NA | | NA |
| N111 | HC | 51 | 19.53 | Male | 25.9 | 10.73 | 0.056 | 6 | 38.6 | 3.1 | 272 | NA | NA | NA | | NA |
| N112 | HC | 55 | 24.49 | Male | 25 | 4.96 | 0.029 | 6.4 | 38.2 | 3.4 | 347 | NA | NA | NA | | NA |
| N114 | HC | 56 | 25.25 | Male | 28.3 | 1.61 | 0.051 | 7.9 | 30.8 | 4.8 | 277 | NA | NA | NA | | NA |
| N59 | HC | 23 | 19.72 | Male | 25 | 11.31 | 0.018 | 5.17 | 34.8 | 3.01 | 294 | NA | NA | NA | | NA |
| N71 | HC | 43 | 20.96 | Male | 25 | 7.22 | 0.048 | 5 | 27.1 | 3.3 | 234 | NA | NA | NA | | NA |
| N72 | HC | 23 | 21.26 | Male | 26.65 | 6.12 | 0.051 | 5.9 | 35.1 | 3.2 | 242 | NA | NA | NA | | NA |
| N73 | HC | 21 | 22.03 | Male | 25 | 1.11 | 0.019 | 5.5 | 33.8 | 2.9 | 228 | NA | NA | NA | | NA |
| N76 | HC | 42 | 24.22 | Male | 25 | 7.95 | 0.024 | 6.3 | 21.2 | 4.6 | 266 | NA | NA | NA | | NA |
| N80 | HC | 40 | 23.82 | Male | 26.8 | 5.55 | 0.015 | 10.6 | 36.6 | 5.59 | 246 | NA | NA | NA | | NA |
| N84 | HC | 47 | 23.56 | Male | 26.95 | 3.74 | 0.028 | 6.52 | 30.8 | 4.14 | 241 | NA | NA | NA | | NA |
| N87 | HC | 46 | 27.44 | Male | 30 | 7.74 | 0.025 | 7.24 | 42.34 | 3.63 | 346.4 | NA | NA | NA | | NA |
| N89 | HC | 39 | 25.31 | Male | 27.15 | 8.02 | 0.001 | 5.3 | 41 | 2.5 | 181 | NA | NA | NA | | NA |
| N91 | HC | 45 | 24.39 | Male | 25 | 3.96 | 0.019 | 5.1 | 24.3 | 3 | 225 | NA | NA | NA | | NA |
| N92 | HC | 57 | 23.66 | Male | 25 | 7.1 | 0.005 | 4.9 | 40.2 | 2.5 | 195 | NA | NA | NA | | NA |
| N95 | HC | 49 | 21.48 | Male | 29.85 | 6.23 | 0.005 | 6.1 | 30.7 | 3.5 | 261 | NA | NA | NA | | NA |
| N97 | HC | 27 | 31.24 | Male | 28.2 | 0.25 | 0.019 | 4.5 | 37.9 | 2.6 | 192 | NA | NA | NA | | NA |
| N99 | HC | 30 | 25.56 | Male | 25.1 | 4.75 | 0.016 | 6 | 30.4 | 3.9 | 326 | NA | NA | NA | | NA |
| SP1-1 | SP | 33 | 21.23 | Male | 87.9 | 8.38 | 0.009 | 4.32 | 50.08 | 2.16 | 447.8 | 2 | Normal | No | | Normal |
| SP1-10 | SP | 46 | 22.86 | Male | 389.35 | 12.08 | 0.026 | 7.3 | 40.51 | 3.87 | 375 | 2 | Lower | No | | Normal |
| SP1-11 | SP | 42 | 21.63 | Male | 25 | 10.5 | 0.02 | 7.39 | 45.61 | 2.83 | 223 | 2 | Lower | Yes | | More |
| SP1-12 | SP | 46 | 18.76 | Male | 28.45 | 9.91 | 0.028 | 13.61 | 22.94 | 10.32 | 355 | 1 | Lower | Yes | | Normal |
| SP1-13 | SP | 48 | 22.09 | Male | 163.15 | 10.51 | 0.012 | 6.46 | 52.31 | 2.5 | 307 | 3 | Lower | No | | Normal |
| SP1-14 | SP | 23 | 21.59 | Male | 67.5 | 12.25 | 0.02 | 4.78 | 50.41 | 1.99 | 300 | 2 | Lower | Yes | | Normal |
| SP1-15 | SP | 44 | 19.94 | Male | 48.985 | >20 | 0.031 | 6.73 | 30 | 4.22 | 428.1 | 3 | Lower | No | | More |
| SP1-16 | SP | 34 | 22.72 | Male | 26.1 | 3.87 | 0.046 | 10.63 | 29.8 | 6.68 | 354 | 3 | Lower | Yes | | Normal |
| SP1-3 | SP | 32 | 23.03 | Male | 202.65 | 14.13 | 0.056 | 12 | 24.7 | 8.51 | 449.9 | 2 | Lower | No | | Normal |
| SP1-6 | SP | 43 | 23.88 | Male | 86.7 | 10.31 | 0.007 | 5.98 | 59.51 | 2.08 | 317 | 1 | Lower | Yes | | Normal |
| SP1-7 | SP | 42 | 22.66 | Male | 31.975 | 5.51 | 0.038 | 10.73 | 26.24 | 5.69 | 373 | 2 | Lower | No | | More |
| SP1-8 | SP | 20 | 27.17 | Male | 34 | 10.38 | 0.034 | 5.04 | 61.71 | 1.6 | 514.1 | 1 | Lower | Yes | | More |
| SP2-10 | SP | 47 | 22.86 | Male | 47.885 | 14.87 | 0.051 | 6.74 | 42.14 | 3.38 | 390 | 3 | Lower | Yes | | Normal |
| SP2-11 | SP | 43 | 21.63 | Male | 46.915 | 15.66 | 0.045 | 8.52 | 43.84 | 3.47 | 266 | 3 | Lower | Yes | | More |
| SP2-13 | SP | 49 | 22.55 | Male | 49.3 | 15.75 | 0.049 | 8.71 | 30.04 | 5.59 | 329 | 3 | Normal | No | | More |
| SP2-15 | SP | 45 | 28.52 | Male | 45.095 | 10.91 | 0.043 | 8.75 | 21.6 | 6.51 | 445.1 | 3 | Normal | No | | Normal |
| SP2-2 | SP | 37 | 24.86 | Male | 47.885 | 9.43 | 0.02 | 6.39 | 39.64 | 2.92 | 473 | 2 | Lower | Yes | | Normal |
| SP2-3 | SP | 48 | 23.12 | Male | 25 | 11.87 | 0.051 | 5.99 | 46.4 | 3.09 | 451 | 2 | Lower | Yes | | More |
| SP2-7 | SP | 43 | 25.25 | Male | 66 | 9.62 | 0.045 | 9.56 | 37.6 | 5.38 | 451 | 1 | Lower | Yes | | More |
| SP2-8 | SP | 21 | 27.17 | Male | 406.95 | 10.73 | 0.038 | 8.11 | 34.6 | 4.65 | 501 | 1 | Lower | Yes | | Normal |
| SP3-13 | SP | 50 | 22.35 | Male | 25 | 12.63 | 0.054 | 7.83 | 41.91 | 3.97 | 506.1 | 3 | Lower | No | | More |
| SP3-17 | SP | 62 | 22.22 | Female | 31.865 | 11.48 | 0.054 | 9.02 | 28 | 6.1 | 389 | 1 | Lower | No | | More |
| SP3-18 | SP | 33 | 18.49 | Female | 413.9 | >20 | 0.037 | 7.82 | 45.41 | 3.4 | 700.1 | 1 | Lower | No | | More |
| SP3-2 | SP | 38 | 24.86 | Male | 489.85 | 11.1 | 0.028 | 7.76 | 34.34 | 4.02 | 503.1 | 1 | Lower | Yes | | Normal |
| SP3-20 | SP | 35 | 24.45 | Male | 422.5 | 9.59 | 0.044 | 6.26 | 46.21 | 2.87 | 428.1 | 3 | Normal | Yes | | Normal |
| SP3-21 | SP | 36 | 24.8 | Male | 623.5 | 5.93 | 0.006 | 8.2 | 31.1 | 5 | 406.1 | 1 | Normal | Yes | | Normal |
| SP3-22 | SP | 67 | 20.9 | Male | 279.3 | 15.76 | 0.016 | 6.3 | 46.44 | 2.9 | 336 | 1 | Normal | Yes | | Normal |
| SP3-23 | SP | 48 | 44.98 | Male | 25 | 10.08 | 0.018 | 5.18 | 48.54 | 2.17 | 285.4 | 3 | Lower | No | | Normal |
| SP3-24 | SP | 66 | 22.66 | Male | 423.35 | >20 | 0.037 | 9.89 | 35.1 | 5.88 | 420.1 | 1 | Lower | Yes | | More |
| SP3-3 | SP | 33 | 23.03 | Male | 25 | 14.71 | 0.03 | 5.18 | 48.54 | 2.17 | 285.4 | 2 | Lower | No | | Normal |
| SP3-7 | SP | 44 | 25.25 | Male | 517 | 12.61 | 0.001 | 6.55 | 34.74 | 3.78 | 410.1 | 3 | Lower | No | | More |
| SP3-8 | SP | 21 | 27.17 | Male | 147.35 | >20 | 0.024 | 11.37 | 36.8 | 6.58 | 509.1 | 1 | Lower | No | | More |

**Table S2.** Significant discrepancy of bacteria taxa on phylum, family, and genus level

|  |  | SP | HC | *P* | P_fdr_ |
| --- | --- | --- | --- | --- | --- |
| Phylum | Firmicutes | 79.17 ± 12.74 | 59.86 ± 17.38 | <0.001 | <0.001 |
|  | Bacteroidetes | 8.22 ± 9.41 | 29.76 ± 20.41 | <0.001 | <0.001 |
| Family | Lachnospiraceae | 53.36±21.44 | 70.23±15.82 | <0.001 | <0.001 |
|  | Bacteroidaceae | 30.26±25.72 | 6.42±9.88 | <0.005 | <0.005 |
|  | Peptostreptococcaceae | 3.47±5.41 | 7.75±8.2 | <0.001 | <0.05 |
|  | Erysipelotrichaceae | 1.93±2.71 | 7.89±6.7 | <0.001 | <0.001 |
|  | Acidaminococcaceae | 7.17±8.41 | 0.57±0.91 | <0.001 | <0.05 |
|  | Coriobacteriaceae | 2.7±4.89 | 4.23±4.33 | <0.001 | <0.05 |
|  | Clostridiaceae_1 | 1.04±1.92 | 2.6±3.94 | <0.005 | <0.05 |
|  | Family_XIII | 0.07±0.13 | 0.31±0.46 | <0.001 | <0.005 |
| Genus | Blautia | 3.02 ± 3.58 | 14.31 ± 8.08 | <0.001 | <0.001 |
|  | [Eubacterium]_hallii_group | 0.66 ± 1.03 | 3.99 ± 2.36 | <0.001 | <0.001 |
|  | Dorea | 0.86 ± 1.01 | 3.43 ± 2.35 | <0.001 | <0.001 |
|  | Lachnospiraceae_FCS020_group | 0.03 ± 0.06 | 0.2 ± 0.18 | <0.001 | <0.001 |
|  | [Ruminococcus]_gauvreauii_group | 0.08 ± 0.22 | 0.55 ± 0.58 | <0.001 | <0.001 |
|  | Unclassified_Lachnospiraceae | 0.7 ± 0.62 | 1.75 ± 1.28 | <0.001 | <0.001 |
|  | Coprococcus_1 | 0.09 ± 0.14 | 0.32 ± 0.3 | <0.001 | <0.005 |
|  | Unclassified_Firmicutes | 0 ± 0 | 0.04 ± 0.07 | <0.001 | <0.005 |
|  | Erysipelotrichaceae_UCG-003 | 0.36 ± 0.56 | 2.53 ± 3.43 | <0.001 | <0.005 |
|  | Anaerostipes | 0.69 ± 0.91 | 2.56 ± 2.87 | <0.001 | <0.005 |
|  | Bacteroides | 16.74 ± 17.07 | 3.97 ± 6.32 | <0.001 | <0.005 |
|  | Turicibacter | 0.02 ± 0.07 | 0.64 ± 1.08 | <0.001 | <0.05 |
|  | [Ruminococcus]_torques_group | 1.38 ± 1.62 | 3.85 ± 4.26 | <0.005 | <0.05 |
|  | Marvinbryantia | 0.02 ± 0.05 | 0.16 ± 0.24 | <0.005 | <0.05 |
|  | Intestinibacter | 0.17 ± 0.29 | 1.67 ± 2.81 | <0.005 | <0.05 |
|  | Phascolarctobacterium | 3.82 ± 5.69 | 0.36 ± 0.57 | <0.005 | <0.05 |
|  | Lachnoclostridium | 2.67 ± 3.7 | 0.44 ± 0.55 | <0.005 | <0.05 |
|  | Terrisporobacter | 0.03 ± 0.05 | 0.37 ± 0.68 | <0.005 | <0.05 |
|  | Clostridium_sensu_stricto_1 | 0.32 ± 0.51 | 1.5 ± 2.41 | <0.005 | <0.05 |
|  | Allisonella | 0.31 ± 0.53 | 0.02 ± 0.04 | <0.005 | <0.05 |
